# Glutamate and Opioid Antagonists Modulate Dopamine Levels Evoked by Innately Attractive Male Chemosignals in the Nucleus Accumbens of Female Rats

**DOI:** 10.3389/fnana.2017.00008

**Published:** 2017-02-23

**Authors:** María-José Sánchez-Catalán, Alejandro Orrico, Lucía Hipólito, Teodoro Zornoza, Ana Polache, Enrique Lanuza, Fernando Martínez-García, Luis Granero, Carmen Agustín-Pavón

**Affiliations:** ^1^Departament de Farmàcia, Tecnologia Farmacèutica i Parasitologia, Universitat de ValènciaValència, Spain; ^2^Departament de Biologia Cel⋅lular, Biologia Funcional i Antropologia Física, Universitat de ValènciaValència, Spain; ^3^Unitat Predepartamental de Medicina, Universitat Jaume ICastelló de la Plana, Spain

**Keywords:** mesolimbic system, olfactory system, pheromones, reward, sexual attraction

## Abstract

Sexual chemosignals detected by vomeronasal and olfactory systems mediate intersexual attraction in rodents, and act as a natural reinforcer to them. The mesolimbic pathway processes natural rewards, and the nucleus accumbens receives olfactory information via glutamatergic projections from the amygdala. Thus, the aim of this study was to investigate the involvement of the mesolimbic pathway in the attraction toward sexual chemosignals. Our data show that female rats with no previous experience with males or their chemosignals display an innate preference for male-soiled bedding. Focal administration of the opioid antagonist β-funaltrexamine into the posterior ventral tegmental area does not affect preference for male chemosignals. Nevertheless, exposure to male-soiled bedding elicits an increase in dopamine efflux in the nucleus accumbens shell and core, measured by microdialysis. Infusion of the opioid antagonist naltrexone in the accumbens core does not significantly affect dopamine efflux during exposure to male chemosignals, although it enhances dopamine levels 40 min after withdrawal of the stimuli. By contrast, infusion of the glutamate antagonist kynurenic acid in the accumbens shell inhibits the release of dopamine and reduces the time that females spend investigating male-soiled bedding. These data are in agreement with previous reports in male rats showing that exposure to opposite-sex odors elicits dopamine release in the accumbens, and with data in female mice showing that the behavioral preference for male chemosignals is not affected by opioidergic antagonists. We hypothesize that glutamatergic projections from the amygdala into the accumbens might be important to modulate the neurochemical and behavioral responses elicited by sexual chemosignals in rats.

## Introduction

Chemical signals detected by the vomeronasal and olfactory systems are key for social communication and sexual advertisement in rodents (Brennan and Kendrick, 2006; Martínez-García et al., 2009). In particular, sexual chemosignals promote strong intersexual attraction, and can be used to condition a place preference in species like mice and hamsters, i.e., they are reinforcing (Martínez-Ricós et al., 2007; Roberts et al., 2012; Bell et al., 2013).

The mesolimbic dopaminergic system has long been implicated in the control of reward-directed, motivated behaviors (Salamone and Correa, 2012), and addiction (Cameron et al., 2014). Thus, both natural reinforcers, such as food and sex, and drugs of abuse activate the mesolimbic pathway and induce dopamine (DA) release from the projections of the ventral tegmental area (VTA) into the nucleus accumbens (Acb) (Bassareo and Di Chiara, 1999; Cheng et al., 2003; Cameron et al., 2014). Since sexual chemosignals are natural reinforcers, they might be able to induce DA release in the Acb. In fact, exposure to female odors and stimulation of the accessory olfactory system induced an increase in DA levels in the Acb of male rats (Louilot et al., 1991; Mitchell and Gratton, 1992).

Anatomical data suggest that circuits conveying olfactory and vomeronasal information might interact with the mesolimbic system to control the behavior elicited by sexual chemosignals (Novejarque et al., 2011; Gutiérrez-Castellanos et al., 2014). In this sense, the Acb is innervated by the amygdala (Novejarque et al., 2011; Pardo-Bellver et al., 2012; Gutiérrez-Castellanos et al., 2014), a structure involved in encoding the affective value of emotional stimuli (Morrison and Salzman, 2010). The amygdala receives both olfactory and vomeronasal inputs, which are direct to its cortico-medial and indirect to its basolateral divisions, respectively (Pitkänen, 2000; Cádiz-Moretti et al., 2016). Data from studies analyzing the expression of immediate-early genes after exposure to opposite-sex odors show that sexual chemosignals are able to activate these neural circuits. For example, exposure to non-volatile male chemosignals increased Fos in the medial amygdala (Me) and medial shell of the Acb (AcbSh) in chemically naïve female mice, whereas exposure to volatile male odors in females that had 4-day experience with male-soiled bedding increased Fos in the basolateral amygdala (BLA) and VTA (Moncho-Bogani et al., 2005). By contrast, Fos was increased in the Me and the core of the Acb (AcbC) in sexually experienced female rats exposed to male-soiled bedding (Hosokawa and Chiba, 2007). Finally, in male rats, estrous female odors increased Fos immunoreactivity in the Me, AcbSh, AcbC, and in the VTA (Kippin et al., 2003; Hosokawa and Chiba, 2005).

Furthermore, selective 6-hydroxidopamine (6-OHDA) lesions targeting the dopaminergic inputs into the anteromedial Acb and olfactory tubercle (OT), disrupted the preference of female mice for male chemosignals (DiBenedictis et al., 2014). By contrast, preference of female mice toward male soiled-bedding was unaffected by 6-OHDA lesions of the dopaminergic somata in the VTA or their projections to the medial Acb (Martínez-Hernández et al., 2006, 2012). Moreover, pharmacological blockade of dopaminergic transmission by systemic injection of DA antagonists did not affect the innate preference of female mice for male chemosignals nor the induction of conditioned place preference to them (Agustín-Pavón et al., 2007). Thus, the contribution of mesolimbic DA to the processing of sexual chemosignals is complex, and it is likely dependent on the regulation of dopaminergic terminals in the Acb rather than on the activity of VTA neurons.

The aim of this study was to explore this possible contribution of the mesolimbic dopaminergic pathway to the processing of sexual chemosignals in female rats. To characterize the response of female rats to male chemosignals, we first checked whether females raised in the absence of males and their odors (chemically naïve females) innately preferred male over female chemosignals, as was previously demonstrated in female mice (Moncho-Bogani et al., 2002). In addition, we tested the effect of focal injections of the opioid antagonist β-funaltrexamine in the posterior VTA (pVTA) on this behavior. The VTA is anatomically and functionally heterogeneous, and animals self-administer addictive drugs more readily in its posterior than in its anterior part (Zangen et al., 2002; Rodd et al., 2005; Ikemoto et al., 2006), suggesting an involvement of the pVTA in reinforcement processes. Dopaminergic neurons in the VTA are controlled by GABAergic neurons, which in turn are inhibited by activation of μ-opioid receptors (Johnson and North, 1992; Jalabert et al., 2011). Moreover, the activation of μ-opioid receptors in the VTA increases dopaminergic efflux to the Acb (Devine et al., 1993). Since, as noted above, reinforcing stimuli elicit an increase in DA efflux in the Acb, we wondered whether blocking opioid receptors in the VTA could have an effect on the preference for male chemosignals –although previous studies showed no effect of VTA lesions (Martínez-Hernández et al., 2006) or systemic opioid antagonism (Agustín-Pavón et al., 2008) on preference for male chemosignals in female mice.

Second, in light of previous results in male rats (Mitchell and Gratton, 1991), we hypothesized that exposure to male chemosignals would increase DA efflux in the Acb of females. To test this hypothesis, DA efflux in the AcbC and AcbSh of females exposed to male-soiled bedding was measured by microdialysis. The levels of DA in the Acb are increased by excitatory glutamatergic inputs from the amygdala and other cortical regions (Floresco et al., 2001a,b; Howland et al., 2002). Conversely, inhibitory GABAergic neurons can decrease the DA tone, and μ-opioid receptors modulate this action (Johnson and North, 1992; Hipólito et al., 2008). Previous studies showed that the regulation of DA level is different between both regions of the Acb. Thus, Hipólito et al. (2008) showed that activation of μ-opioid agonists in the AcbC enhanced DA levels, whereas the same treatment decreased DA levels in the AcbSh. Therefore, we tested whether an opioid antagonist (naltrexone) would blunt the DA response in the AcbC. On the other hand, it has been shown that blocking NMDA receptors in the AcbSh decreases DA efflux upon stimulation of the BLA (Howland et al., 2002). Hence, we checked the effect of a glutamate antagonist (kynurenic acid) in the DA efflux elicited by male chemosignals in the AcbSh.

## Materials and Methods

### Animals

For this study we used 66 female Wistar rats, aged more than 12 weeks of age. To obtain chemically naïve female rats, females were reared in the absence of mature males or their derived chemicals signals (Moncho-Bogani et al., 2002). Briefly, we housed pregnant females in a room without male rats, sexed the litters and separated the male siblings 19 days after delivery, early before puberty. Experimental females were housed in the same room without males, so they were both sexually and chemically inexperienced. A previous study in mice showed that ovariectomized females treated with either oil, estradiol or estradiol + progesterone displayed similar levels of innate attraction toward male chemosignals, while showing the expected differences in receptivity to a stud male (Moncho-Bogani et al., 2004). Thus, we deemed unnecessary to track the phase of the estral cycle of the rats.

Rats were housed in plastic cages (48 cm × 38 cm × 21 cm) in groups of four to six, with controlled humidity and temperature (22°C), a 12:12-h light/dark cycle, and water and food available *ad libitum*. All the procedures were carried out in strict accordance with the EEC Council Directive 86/609, Spanish laws (RD 53/2013) and animal protection policies. The protocols were approved by the Animal Care Committee of the Faculty of Pharmacy at the University of Valencia, Spain.

### Drugs

The irreversible antagonist of the μ-opioid receptor, β-funaltrexamine, and the broad-spectrum antagonist of the opioid receptors, naltrexone, were obtained from Tocris (Bristol, UK). Kynurenic acid, an antagonist of NMDA, AMPA and kainate glutamate receptors, was obtained from Sigma–Aldrich Co. Stock solutions of the drugs were prepared by dissolving the compound in the proper volume of distilled water. These solutions were aliquoted and kept frozen at -40°C until use. Prior to use, aliquots of the stock solutions were conveniently diluted in artificial cerebrospinal fluid solution (aCSF) (Sánchez-Catalán et al., 2009).

### Surgery

Rats were anesthetized with 95 mg/kg of ketamine plus 10 mg/kg of xylazine intraperitoneally (i.p.) and placed in a stereotaxic apparatus (Stoelting, USA). An incision (8–10 mm) was made in the skin above the skull and the wound margin was infiltrated with lidocaine (3%).

Animals for Experiment 1b were implanted unilaterally with a 28-gage guide cannula (Plastics One, USA) aimed at 1.0 mm above the pVTA. A stainless steel stylet (33-gauge) extending 1.0 mm beyond the tip of the guide cannula was introduced at the time of surgery and removed at the time of testing. After surgery, rats were left to recover for 3 days before the experiment. For Experiments 2 and 3, animals were implanted with one concentric microdialysis probe with 2 mm of permeable membrane (Hospal, AN69) in the AcbC (Experiment 2) or the AcbSh (Experiment 3). The brain coordinates related to bregma and skull surface were: pVTA, A/P: -6.0 mm, L: -2.1 mm, V: 7.9 mm (10° from the vertical midline); AcbC, A/P: +1.3 mm, L: -1.4 mm, V: -8.1 mm; AcbSh, A/P: +1.3 mm, L: -0.8 mm, V: -8.3 mm, according to Paxinos and Watson (2007).

### Microdialysis and Analytical Procedures

Dialysis experiments were performed 24 h after surgery and rats were used for only one experiment. The use of this recovery period was shown to be sufficient in several previous published studies (Santiago and Westerink, 1990; Santiago et al., 2000; Hipólito et al., 2008, 2009a,b). PE10 inlet tubing was attached to a 2.5 mL syringe (Hamilton), mounted on a syringe pump (Harvard Instruments, South Natick, MA, USA) and connected to the dialysis probes that were perfused at 3.5 μL/min with aCSF solution. Fractions of dialysate were on-line analyzed for DA content every 20 min using an HPLC system with electrochemical detection, as previously described (Hipólito et al., 2008). The HPLC system consisted of a Waters 510 series pump in conjunction with an electrochemical detector (Mod. Intro, Antec, Leyden, The Netherlands). The applied potential was +0.55V (vs. Ag/AgCl). Dialysates were injected onto a 5 mm RP-18 column (LiChroCART 125-4, Merck, Darmstadt, Germany) via a VALCO valve fitted with a 65 μL sample loop. The mobile phase consisted of a sodium acetate/acetic acid buffer (Hipólito et al., 2008), which was pumped through the column at a flow rate of 0.2 mL/min. Chromatograms were integrated and compared with separately run standards on each experimental day, using the AZUR 4.2 software (Datalys, France). Detection limit was defined by a signal to noise ratio of 2:1, being approximately 6 fmol/sample.

### Experiments

#### Experiment 1A: Innate Preference of Female Rats for Male Chemosignals

To investigate whether female rats display an innate preference for male chemosignals, 15 sexually inexperienced and chemically naïve females underwent a two-choice test. These tests were performed in rectangular clear methacrylate cages (25 cm × 50 cm × 45 cm) with two glass dishes (6 cm × 5.5 cm), containing clean or soiled bedding, located on opposite sides of the cage, following the protocol by Martínez-Ricós et al. (2007). Female-soiled bedding was obtained from home cages containing 3–6 female rats of the same strain for 4 days, whereas male-soiled bedding was collected from dominant males individually housed, mixed and homogenized, as previously described (Martínez-Ricós et al., 2007). Bedding was stored at -20°C until the day of the test.

On the 1st and the 2nd day, female rats were placed in the test cages containing two dishes of clean bedding for 5 min for habituation. On the 3rd day, a control test was run, with the two dishes containing female-soiled bedding, and their behavior was video recorded for 5 min. Since we were aiming to an unbiased two-choice test, rats that spent twice as much time exploring one of the dishes than the other in the control test were discarded for further analysis (*n* = 6). On the 4th day, females were placed in the test cages with one of the dishes containing female-soiled bedding and the other dish containing male-soiled bedding (male preference test), and their behavior was recorded for 5 min.

#### Experiment 1B: Effect of μ-Opioid Antagonism in the pVTA on the Preference for Male Chemosignals

To test whether opioid antagonism in the pVTA would affect the behavioral preference of females toward male chemosignals, rats were implanted with a cannula in the pVTA (see above, *n* = 10). The use of unilateral injections of β-funaltrexamine in the pVTA was effective in previous studies in blocking the locomotor-stimulant effects of ethanol and its metabolites (Sánchez-Catalán et al., 2009; Hipólito et al., 2010). Thus, the use of bilateral injections was deemed unnecessary. Habituation to the experimenter and the injection procedure consisted of 4 days of handling for 5 min/day, starting 3 days after surgery. Seven days after surgery, female rats were tested in a two-choice test as described in Experiment 1A. Rats that showed a biased investigation of the test cage were discarded (*n* = 1). Rats were intra-pVTA administered with β-funaltrexamine (2.5 nmol) (Sánchez-Catalán et al., 2009) after the control test, (female vs. female-soiled bedding, 3rd day). The microinjection of β-funaltrexamine into the pVTA was made via 33-gage stainless steel injectors extending 1.0 mm below the tip of the guide cannula. The injector was attached to a 25 μL Hamilton syringe by using PE-10 tubing and located on an infusion pump programmed to deliver a volume of 300 nL. The injector remained in place for 1 min and, then, it was replaced by the stylet. The use of an irreversible antagonist allowed to perform the microinjection the day before the preference test, ensuring the μ-opioid receptors blockade in the pVTA and avoiding the possible stress of the animals due to the microinjection procedure. Animals were tested 24 h after the drug injection as in Experiment 1A.

#### Experiment 2: DA Efflux in AcbC Elicited by Male Chemosignals and Effect of Opioid Antagonism

This experiment was designed to investigate whether DA efflux was increased by male chemosignals in the AcbC. We also investigated the possible effect of opioid antagonists in this neurochemical response. Females were implanted with one concentric microdialysis probe into the AcbC as described in section “Surgery.” Experiments were performed 24 h after surgery. Female rats were located in a rectangular cage (25 cm × 50 cm × 45 cm) containing a dish with clean bedding. The dose and time of administration of naltrexone (100 μM) was selected by means of dose-response experiments to ensure that it did not affect DA baseline levels (data not shown). The application of naltrexone or aCSF by reverse dialysis through the microdialysis probe was initiated after the establishment of the DA baseline, and it was maintained until the end of the experiment. Twenty minutes after the third baseline point, the dish containing clean bedding was substituted by another dish containing either clean or male-soiled bedding. Rats were allowed to explore the new dish for 40 min, and then the dish was substituted again by the initial one containing clean bedding. The dialysis procedure continued for 100 min. We assessed the possible change in DA efflux by the manipulation of the dish containing clean bedding in two groups of rats (vehicle + clean bedding, *n* = 6; naltrexone + clean bedding *n* = 7). Second, we measured the change in DA efflux in the AcbC induced by male chemosignals and whether this change was affected by naltrexone in two additional groups of females (vehicle + male-soiled bedding, *n* = 7; naltrexone + male-soiled bedding; *n* = 7).

#### Experiment 3: DA Efflux in AcbSh Elicited by Male Chemosignals and Effect of Glutamate Antagonism

To check whether DA efflux was elicited by male chemosignals in the AcbSh, females were implanted with a concentric microdialysis probe in this region as in Experiment 2. In addition, we investigated whether the neurochemical response was modulated by glutamate antagonism. Females were randomly assigned to two experimental groups (vehicle + male-soiled bedding, *n* = 8; kynurenic acid + male-soiled bedding, *n* = 6). The procedure was identical to Experiment 2, except that male bedding was introduced 10 min after the third baseline and kynurenic acid application by reverse dialysis was maintained for 80 min only. The dose and time of the kynurenic acid administration (50 μM) was selected by means of dose-response experiments to ensure that it did not affect DA baseline levels, as above (data not shown). We recorded the behavior of these animals to analyze the time they spent investigating the male-soiled bedding during the 40 min of exposure. For the vehicle group, the DA sample from two animals and the behavioral recording of two other animals could not be obtained. Thus, we could analyze *n* = 6 for each measurement in this group.

### Behavioral Measures

Experiments were recorded using a video camera. For Experiment 1, we automatically measured the time that females spent in a defined area (6 cm × 5.5 cm) by means of the video tracking software Raddot (University of Valencia, Spain), during the 5 min of the test. Furthermore, an experimenter who was blind to the treatment of the animal and type of bedding measured the time that the animal spent digging on the bedding contained on the dish using a stopwatch. Previous data showed that female mice spent a significantly longer proportion of time digging on male-soiled bedding than in female-soiled bedding (Agustín-Pavón et al., 2007). Thus, digging was taken as an approximate measure of the attractive value of the stimulus for the animal. For Experiment 3, a person who was unaware of the treatment recorded by means of a stopwatch the time in seconds that each rat spent investigating the dish, i.e., the time that females spent sniffing and digging on the male-soiled bedding, as a measure of exposure to the olfactory and vomeronasal cues contained in the it.

### Histology

**FIGURE 1:**
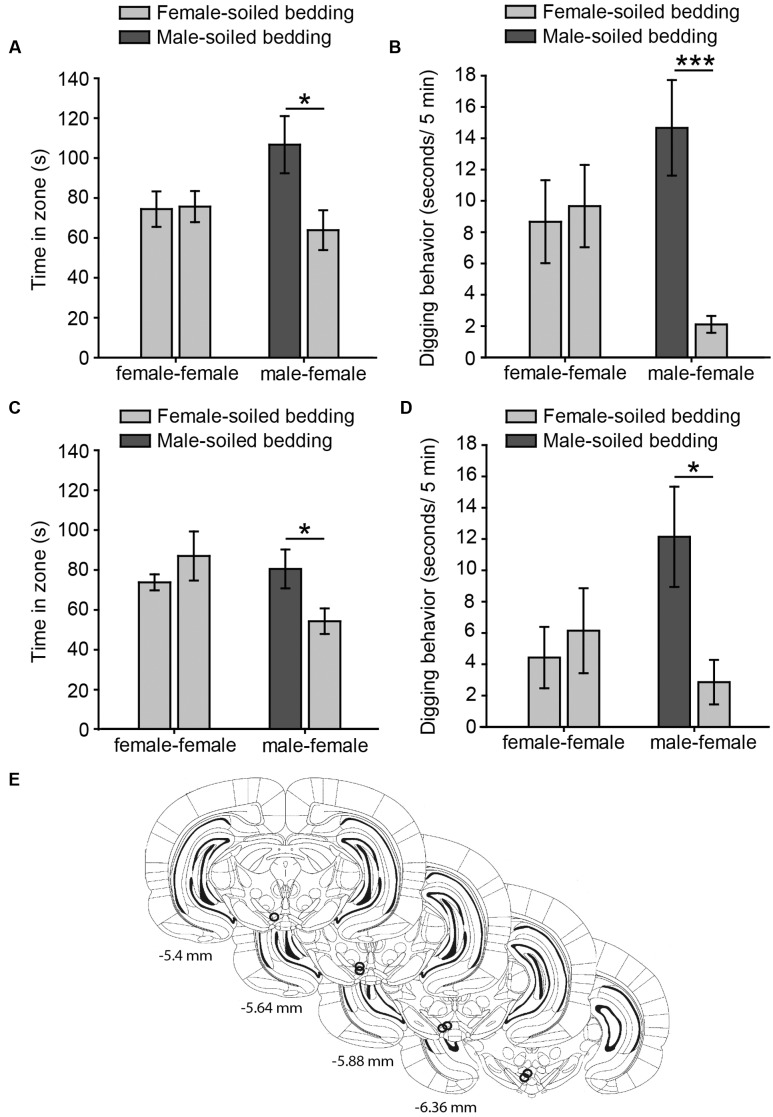
**Virgin female rats display an innate preference for male-soiled bedding in two-choice tests, which is not affected by intra-pVTA microinjection of a μ-opioid antagonist.** The bar charts represent the time spent by females in each zone **(A,C)** and time digging on the dishes **(B,D)** containing female-soiled bedding in the control session (female-female, light gray bars) and in the test (male-female, dark gray and light gray bars, respectively). Females spent significantly more time in the zone and dug significantly more on male-soiled bedding than on female-soiled bedding **(A,B)**, and these behaviors were not affected by focal injections of β-funaltrexamine in the pVTA **(C,D)**. **(E)** Diagram of coronal sections of the brains of experimental subjects depicting the placement of the tip of the injection cannulae in the pVTA, represented by circles, where the stainless steel injector was extended, and therefore, the pharmacological solution was injected. Numbers indicate distance to bregma in mm, adapted from Paxinos and Watson (2007). ^∗^*p* < 0.05, ^∗∗∗^*p* < 0.001. Data are represented as mean ± SEM.

**FIGURE 2:**
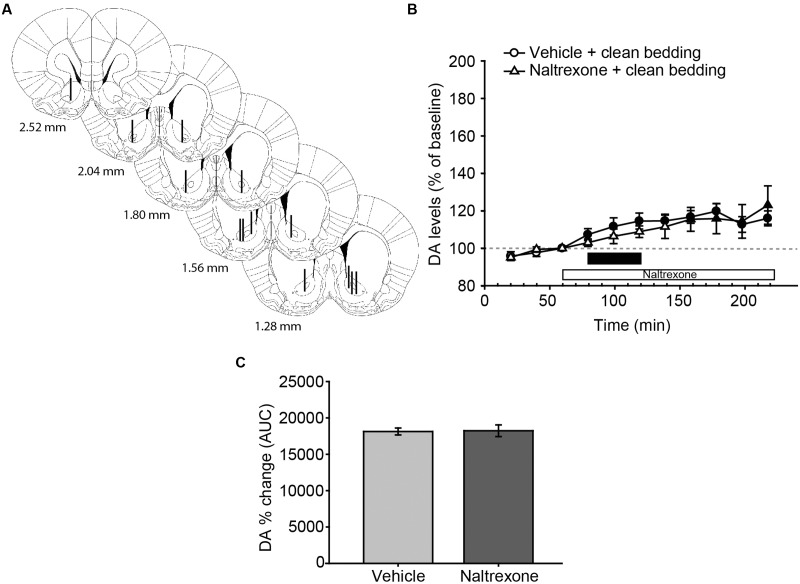
**Exposure to a new dish with clean bedding elicits a moderate increase in DA efflux in the AcbC, which is not affected by treatment with naltrexone.** **(A)** Diagram of coronal sections of the brains of experimental rats, indicating the placement of microdialysis probe in the AcbC. The location of the probes in vehicle-treated animals is represented in the left hemisphere, and in the naltrexone-treated animals in the right hemisphere. The vertical lines represent the length of the active membrane of the probe, where the substances exchange and the dialysate recovery take place. Numbers indicate distance to bregma in mm, adapted from Paxinos and Watson (2007). **(B)** Exposure to a new dish containing clean bedding induces a mild increase in DA efflux with respect to third baseline (filled symbols). Naltrexone treatment does not affect the DA levels in AcbC of female rats exposed to clean bedding. The black bar indicates the period of the bedding exposure and the white bar represents the naltrexone treatment. **(C)** Comparison of DA change (AUC) between vehicle and naltrexone-treated groups confirms that the pharmacological treatment does not affect DA levels. Data are represented as mean ± SEM.

**FIGURE 3:**
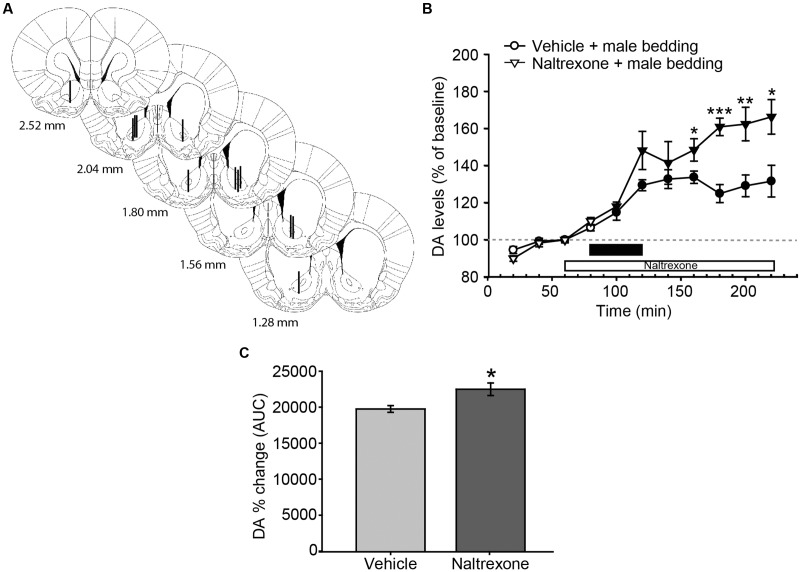
**Exposure to male sexual chemosignals induces a significant and sustained release of DA in the AcbC of female rats, and the blockade of opioid receptors increases the DA efflux with a time delay.** **(A)** Diagram of coronal sections from the brains of rats, indicating the placement of microdialysis probe in the AcbC. The location of the probes in vehicle-treated animals is represented in the left hemisphere, and in the naltrexone-treated animals in the right hemisphere. The vertical lines represent the length of the active membrane of the probe, where the substances exchange and the dialysate recovery take place. Numbers indicate distance to bregma, adapted from Paxinos and Watson (2007). **(B)** Exposure to male chemosignals induces a significant increase of DA levels in the AcbC in vehicle-treated animals (filled circles indicate a significant difference with respect to third baseline), which is further enhanced by the treatment with naltrexone 80 min post-exposure (filled triangles, stars). The black bar indicates the period of the bedding exposure and the white bar represents the naltrexone treatment. **(C)** Comparison of DA change (AUC) between vehicle and naltrexone-treated groups revealed significant differences between treatments. ^∗^*p* < 0.05, ^∗∗^*p* < 0.01, ^∗∗∗^*p* < 0.001, differences between groups. Data are represented as mean ± SEM.

**FIGURE 4:**
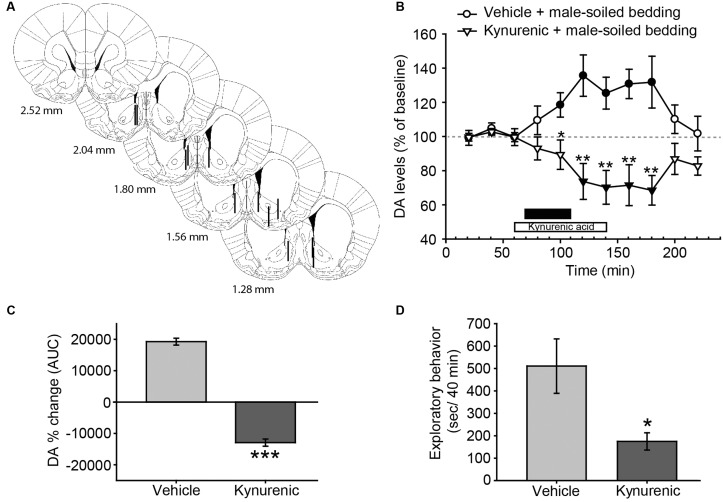
**Treatment with kynurenic acid decreases DA release in the AcbSh and investigation induced by exposure to male-soiled bedding in female rats.** **(A)** Diagram of coronal sections from the brains of rats, indicating the placement of microdialysis probe in the AcbSh. The location of the probes is represented in the left hemisphere for vehicle-treated animals, and in the right hemisphere for kynurenic-treated animals. Numbers indicate distance to bregma, adapted from Paxinos and Watson (2007). **(B)** Exposure to male-soiled bedding induces a significant increase with respect to baseline in the AcbSh that lasts from 20 min until 100 min post-exposure (filled circles represent significant differences with respect to third baseline). Treatment with kynurenic acid decreases DA levels with respect to baseline from 40 to 100 min after exposure (filled triangles). The black bar indicates the period of exposure to male-soiled bedding and the white bar represents the kynurenic acid treatment. **(C)** Comparison of DA change (AUC) between vehicle and kynurenic acid-treated groups reveals significant differences between treatments. **(D)** Bar chart representing time spent by females investigating the dish containing male-soiled bedding in the vehicle-treated and kynurenic acid-treated groups. The treatment with kynurenic acid significantly decreases the time that females spent investigating male chemosignals as compared to vehicle. ^∗^*p* < 0.05, ^∗∗^*p* < 0.01, ^∗∗∗^*p* < 0.001, differences between groups. Data are represented as mean ± SEM.

At the end of the experiments, animals were deeply anesthetized and killed by decapitation. Brains were quickly removed, frozen in isopentane and cut in a cryostat into 40 μm thick coronal sections. The slices were mounted, stained with cresyl violet and evaluated histologically to confirm the position of the cannula tips and the microdialysis probes. Only rats with the cannula tip or probe correctly placed were included in the statistical analysis. The position of the tips of the cannulae and microdialysis probes is depicted in **Figures 1E**, **2A**, **3A**, and **4A**. Representative samples of Nissl-stained coronal sections are provided in **Supplementary Figure 1**.

### Statistical Analysis

Data are represented as mean ± SEM. In Experiment 1, we analyzed the time that females spent in the defined area around the dishes and digging on the bedding in seconds by means of Student’s *t*-tests. In Experiments 2 and 3, the level of DA was expressed as percentage of baseline, defined as 100% DA concentration in the Acb. The effects of treatments and bedding exposure on DA levels were analyzed through a mixed two-way analysis of variance (ANOVA) of repeated measures, with time as within-subject factor and treatment as between-subject factor. This analysis was followed by Dunnet’s *post hoc* test to identify the time points that differed significantly from the respective baseline (third baseline time point). Significant time × treatment interactions were analyzed by *post hoc* analyses with the Bonferroni correction when appropriate. Areas under the curve (AUC) for DA change (%) were calculated from 60 to 220 min and analyzed by means of a Student’s *t*-test. The level of significance was set at *p* < 0.05. All the analyses were performed using SPSS, v. 15.0 (SPSS, Inc., Chicago, IL, USA).

## Results

### Experiment 1: Female Rats Display an Innate Preference for Male over Female Chemosignals, Which Is Not Affected by Focal Injection of β-Funaltrexamine into the pVTA

Our data from Experiment 1a shows that chemically naïve female rats prefer to investigate male- to female-soiled bedding in a two-choice test, suggesting that females display an innate attraction for male chemosignals. Thus, the time that females spent around both dishes containing female-soiled bedding was identical in the control (*p* = 0.92), whereas females spent significantly more time around male-soiled bedding than around female-soiled bedding in the male preference test (*p* = 0.026) (**Figure 1A**). In addition, rats spent more time digging on male-soiled bedding as compared to female-soiled bedding (*p* = 0.0009) (**Figure 1B**). Moreover, the proportion of time digging on the bedding with respect to the time spent in each zone was significantly higher for male-soiled bedding (female-soiled bedding = 4.16 ± 1.27%, male-soiled bedding = 15 ± 2.8%, *p* = 0.003).

For Experiment 1B, we first evaluated the cannulae placements and animals with the tip of the cannula in the pVTA were included in the statistical analysis (**Figure 1E**). Female rats treated with β-funaltrexamine spent more time in the male zone (*p* = 0.044, **Figure 1C**) and digging on male-soiled bedding (*p* = 0.02, **Figure 1D**) than in the female zone, whereas no differences were observed in the control test, neither in time spent in the zone nor in digging (*p* > 0.1 in both cases) (**Figures 1C,D**). Moreover, rats treated with β-funaltrexamine displayed similar percentages of digging on the bedding than non-treated rats of Experiment 1A, and the percentage of digging on male-soiled bedding was significantly higher than on female-soiled bedding (female = 4.90 ± 2.15%, male = 14.14 ± 2.88%, *p* = 0.02). Thus, intra-pVTA microinjection β-funaltrexamine did not affect the attraction of female rats for male chemosignals, suggesting that μ-opioid receptors in the pVTA are not involved in the expression of this innate behavior.

### Experiment 2: Exposure to Male Soiled Bedding Increases DA Efflux in the AcbC, Which Shows a Delayed Enhancement by Naltrexone Administration

Following histological evaluation, animals with correct microdialysis probe placement were included for analysis (**Figures 2A** and **3A**). The statistical analysis of the data revealed that exposure to a new dish with clean bedding elicited a mild increase in DA efflux in the AcbC with respect to baseline (**Figure 2B**, filled symbols). The administration of naltrexone did not affect DA levels after the introduction of a new dish with clean bedding, since the analysis revealed no differences for treatment [*F*_(1,12)_ = 0.146, *p* = 0.709], or the interaction time × treatment [*F*_(10,120)_ = 1.434, *p* = 0.178]. The effect of the new dish was reflected in a significant effect of main factor time [*F*_(10,120)_ = 8.258, *p* < 0.001]. Thus, DA efflux peaked with a 20% increase over baseline 180 min after the onset of the experiment, i.e., 100 min after the first manipulation of the dish (**Figure 2B**). Finally, the comparison of AUCs of DA change revealed no significant differences between vehicle and naltrexone-treated animals after exposure to clean bedding (**Figure 2C**).

Male-soiled bedding evoked a significant increase of DA in the AcbC (**Figure 3B**). The mixed two-way ANOVA revealed statistically significant main effects of time [*F*_(10,110)_ = 46.955, *p* < 0.001] and treatment [*F*_(1,11)_ = 8.349, *p* = 0.015], as well as a significant interaction [time × treatment, *F*_(10,110)_ = 5.759, *p* < 0.001]. In the vehicle-treated animals, DA efflux was significantly increased with respect to baseline 20 min after the introduction of the dish containing male-soiled bedding, and peaked with a 33% increase over baseline 160 min after the onset of the experiment, i.e., 80 min after the introduction of the male-soiled bedding.

We also compared the increase in DA efflux after clean bedding and male soiled bedding exposure in the vehicle-treated groups. The ANOVA revealed a significant effect of the type of bedding, showing that DA levels were significantly higher after the introduction of male-soiled bedding than after the introduction of a new dish with clean bedding [*F*_(1,12)_ = 6.421, *p* = 0.026, compare **Figures 2B** and **3B**].

Furthermore, we explored the differences between vehicle and naltrexone-treated groups exposed to male-soiled bedding. A *post hoc* comparison revealed that DA level in the naltrexone-treated group was higher than in vehicle-treated animals from minute 160 after the onset of the experiment, and this higher level was maintained until the end of the experiment, when DA level peaked in the naltrexone-treated animals with an increase of 66% with respect to baseline (at minute 220, **Figure 3B**). Moreover, the comparison of AUC’s of dopamine change showed that the percentage DA change in the AcbC following male-soiled bedding exposure was higher in the naltrexone-treated animals than in the vehicle group (*p* = 0.014) (**Figure 3C**).

### Experiment 3: Exposure to Male-Soiled Bedding Elicits an Increase in DA Efflux in the AcbSh, Which Is Blocked by Kynurenic Acid Administration

In this experiment, we investigated the possible changes in DA efflux in the AcbSh and its regulation by glutamate receptors. Animals with correct probe placement were included in the analysis (**Figure 4A**). The ANOVA revealed a significant effect of the factor treatment [*F*_(1,10)_ = 16.345, *p* = 0.002] and the interaction between time × treatment [*F*_(10,100)_ = 7.34, *p* < 0.001], but no significant effect of time [*F*_(10,100)_ = 0.597, *p* = 0.813] (**Figure 4B**). *Post hoc* analysis revealed that the exposure to male-soiled bedding increased DA levels over baseline 20 min after the onset of the exposure to bedding in vehicle-treated animals, but returned to baseline before the end of the experiment, 100 min after the onset of exposure to male bedding. By contrast, the treatment with kynurenic acid blocked the DA response and reduced DA levels in the AcbSh (**Figure 4B**). This difference between treatments was additionally confirmed following the comparison of AUC’s of DA change (*p* < 0.001) (**Figure 4C**).

Since kynurenic acid decreased the dopaminergic response in the AcbSh to male chemosignals, we wondered whether this drug had some behavioral effect. To check this, we measured the time that females spent investigating the dish containing male-soiled bedding. A Student’s *t*-test revealed that females treated with kynurenic acid spent significantly less time investigating male bedding than females treated with vehicle (**Figure 4D**). To investigate the dynamics of this reduction, we divided the 40 min of exposure in eight slots of 5 min, and compared these slots between groups. An ANOVA for repeated measures using time slot as within-subject factor revealed a significant main effect of this factor (*F*_7,4_ = 60.860, *p* = 0.001) and also of the between-subject factor group (*F*_1,10_ = 0.025) as well as a significant effect of the interaction time × group (*F*_7,4_ = 12.704, *p* = 0.014). Pairwise comparisons revealed that time spent investigating the male bedding was not significantly different between groups during the first slot (vehicle-treated group, 94.2 ± 12.7 s; kynurenic acid-treated group, 81.6 ± 21.2 s, *p* = 0.623), but became significantly lower in the kynurenic acid-treated animals during the second slot (vehicle-treated group, 99.3 ± 15.9 s; kynurenic acid-treated group, 45.6 ± 17.4 s, *p* = 0.046). Further, time spent investigating by the animals in the vehicle group was similar across the first 35 min, and it declined to 0 only during the last 5 min of exposure. By contrast, the time spent investigating the bedding in the kynurenic acid group declined rapidly, so it was 0 during the last 15 min.

## Discussion

Our results show that chemically naïve female rats, i.e., females that have been raised in complete absence of males and their odors, innately preferred investigating male-soiled bedding, and devoted a higher proportion of time digging on it than on female-soiled bedding. Focal injections of β-funaltrexamine, an irreversible opioid antagonist, in the pVTA, did not affect the behavioral preference of female rats for male chemosignals. Exposure to male chemosignals provoked significant increases in DA efflux in the Acb, which were enhanced by reverse dialysis of naltrexone in the AcbC and abolished by reverse dialysis of kynurenic acid in the AcbSh. Blocking the dopaminergic efflux in the AcbSh by kynurenic acid resulted in an overall decrease of the investigation of sexual chemosignals.

### Male Chemosignals Are Innately Attractive for Female Rats

Sexual behavior is under strict hormonal control in female rodents (Giuliano et al., 2010). However, previous studies showed that both freely cycling and ovariectomized, steroid- or oil-treated female mice, reared in complete absence of male mice, prefer investigating male-soiled bedding (Moncho-Bogani et al., 2002, 2004). These results suggest that intersexual attraction mediated by male chemosignals is innate and might be independent on the hormonal status in female mice. Furthermore, male-soiled bedding was effective as a reinforcer to induce conditioned place preference in freely cycling female mice (Agustín-Pavón et al., 2007; Martínez-Ricós et al., 2007). In the present study, we extend this observation to female rats, showing that chemically naïve, freely cycling adult females innately prefer investigating male chemosignals. This preferential investigation is unlikely to be due to a novelty effect, since a novel neutral odor did not induce preferential investigation in female mice using this protocol (Martínez-Ricós et al., 2007). Moreover, preference for male bedding was persistent for 4 consecutive days, whereas preference for castrated male or female chemosignals disappeared with repeated testing (Martínez-Ricós et al., 2007). Further experiments, however, should be carried out to investigate whether, in female rats, preference for male chemosignals is persistent in consecutive tests.

Sexual receptivity and intersexual attraction mediated by chemosignals seem to be under different regulatory mechanisms. In fact, ovariectomized female mice treated with vehicle or progesterone displayed similar levels of preference for male chemosignals than ovariectomized females primed with estradiol or estradiol + progesterone, although only the latter were receptive in direct encounters with a male, whereas vehicle and progesterone-treated females displayed high levels of refusal behavior (Moncho-Bogani et al., 2004). In this sense, male sexual pheromones can promote effective tracking of a sexual partner enhancing the probability of copulation in the short period of ovulation. In fact, sexual maturation and ovulation are induced by male pheromones in mice (Vandenbergh, 1969), giving adaptive value to the fact that females are attracted by male pheromones independently on their endocrine status. However, other studies do not fully support these results, since progesterone might be inhibitory for pheromone attraction (Dey et al., 2015). It is likely that hormonal regulation is less important in inexperienced females, since both in the present study and in the studies by Moncho-Bogani et al. (2002, 2004) and Martínez-Ricós et al. (2007), females were confronted for the first time with male odors, whereas in the study by Dey et al. (2015) it was not disclosed whether females were raised in the absence of males. Thus, the phase of estral cycle might have an impact in sexually experienced females, but not in our inexperienced females. Further experiments are necessary to test this possibility.

Another key difference relies on the stimuli that were used: the studies showing independency on the hormonal status, including the present one, used male-soiled bedding, containing a wide variety of chemosignals, whereas the study showing the inhibitory effect of progesterone used male urinary proteins (MUPs). Although MUPs alone are sufficient to elicit strong attraction in mice (Roberts et al., 2010), and female rats find those males which excrete a higher proportion of MUPs more attractive (Kumar et al., 2014), other chemosignals may contribute to modulate behavioral preference.

Anyhow, MUPs are detected by the vomeronasal system (Papes et al., 2010; Chamero et al., 2011; Kaur et al., 2014), which is necessary for the display of innate attraction toward male chemosignals, at least in female mice (Martínez-Ricós et al., 2008). The detection of vomeronasal stimuli requires specific behaviors, such as tongue-flick in snakes (Martínez-Marcos et al., 2001) or nuzzling in opossums (Poran et al., 1993), elements that have not been described in mice or rats. In this respect, we found that females dig on male-soiled bedding a significantly higher proportion of the time they spent in the vicinity of male bedding than in female bedding –in particular, the percentage of time females devoted to digging on male-soiled bedding was almost four times higher than the percentage of time digging on female bedding. This result is in agreement with a previous study in mice, showing that the proportion of time that females devoted to digging on male-soiled with respect to total investigation was twofold of the proportion they spent digging on female-soiled bedding (Agustín-Pavón et al., 2007). We hypothesize that digging might be related to the searching and detection of non-volatile, vomeronasal-detected pheromones, since the snout is in the closest contact with the substrate when the animal is digging on it. Further, sniffing opposite-sex chemosignals might be viewed as a reward-seeking behavior (Agustín-Pavón et al., 2007; Malkesman et al., 2010), to which the contribution of the mesolimbic dopaminergic system has been largely known (Salamone and Correa, 2012).

Vomeronasal information reaches the Acb through sparse projections from the posteromedial cortical amygdala (Ubeda-Bañon et al., 2008; Gutiérrez-Castellanos et al., 2014). In addition, olfactory and vomeronasal information are conveyed to the Acb through conspicuous afferents from the BLA (Novejarque et al., 2011). Finally, sparse projections from the medial amygdala reach both the Acb and VTA (Pardo-Bellver et al., 2012).

**FIGURE 5:**
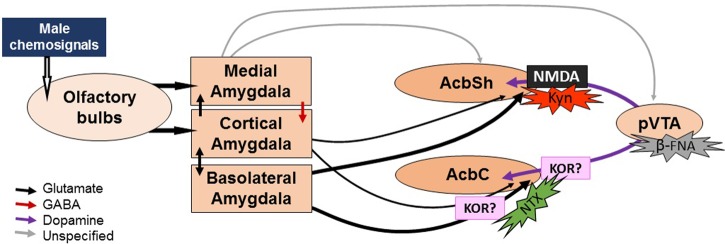
**Sketch of the proposed neural circuit for chemosignal processing and action sites of drug treatments.** Male chemosignals are relayed from the olfactory epithelium and the vomeronasal organ to the olfactory bulbs, which project to the cortical and medial divisions of the amygdala. Then, the olfactory information reaches the Acb via direct projections from these amygdalar divisions, or indirectly through the basolateral division of the amygdala, which is reciprocally connected to the cortical division. Efflux of DA can be elicited without pVTA activation (hence the lack of effect of pharmacological manipulations of the pVTA) by the amygdalar input into the dopaminergic terminals. Kynurenic acid (Kyn) would block the action of the amygdalar input, blocking the release of DA. By contrast, naltrexone (NTX) would not affect the DA efflux during exposure to male chemosignals. We hypothesize that the delayed increase in DA levels might be due to the pharmacological profile of the drug, which might cause a disinhibition of the dopaminergic release via κ-opioid receptors (KOR). The width of the arrows represents the strength of the projections (see Discussion).

Our results show that blocking μ receptors in the pVTA with the irreversible antagonist β-funaltrexamine does not affect preference for male-soiled bedding. Systemic treatment with the opioid antagonist naloxone did not affect the behavioral preference for male chemosignals in female mice (Agustín-Pavón et al., 2008), a result that is in agreement with our data showing that local application of an opioidergic antagonist does not affect the behavioral preference of female rats. The use of unilateral injections of β-funaltrexamine in the pVTA was effective in previous studies in blocking the locomotor-stimulant effects of ethanol and its metabolites/derivatives, while leaving basal locomotion unaffected (Sánchez-Catalán et al., 2009; Hipólito et al., 2010). Moreover, preliminary data in our laboratory have shown that the microinjection of β-funaltrexamine attenuates the stimulating effects of DAMGO, a μ-opioid agonist, into the pVTA throughout 1 week (unpublished results). Likewise, the pretreatment with this antagonist has also been shown to attenuate the effects of several drugs of abuse, remaining this effect for up 3–6 days, depending on the administered site (Ward et al., 2003; Martin et al., 2008). Thus, it could be assumed that the lack of effect shown in the present results reflects a lack of involvement of the opioidergic modulation in the pVTA in the preference for male chemosignals. In fact, innate attraction toward male sexual pheromones is independent from the integrity of the dopaminergic neurons of the VTA in female mice (Martínez-Hernández et al., 2006) and VTA is not activated by the first exposure to male chemosignals as measured by Fos in a study (Moncho-Bogani et al., 2005). We thus hypothesize that pheromonal information might be able to bypass the VTA and be conveyed directly to the Acb via amygdaloid projections (**Figure 5**). However, DiBenedictis et al. (2015) recently showed that male chemosignals, but not female chemosignals, induce c-Fos in the VTA of female mice. Thus, further experiments are needed to reassess the role of the VTA in the preference for male chemosignals.

### Male Chemosignals Elicit Dopaminergic Efflux in the Accumbens

Our results extend to the female rat an older observation made in males showing an increased DA signal in the AcbC of male rats exposed for 20 min to estrous female odors, but not odors from ovariectomized females or males (Mitchell and Gratton, 1991). A close analysis of the results of Mitchell and Gratton (1991) reveals that the increase in DA efflux quickly returned to baseline 5 min after the termination of the exposure, whereas in our study the DA levels did not return to baseline before termination of the experiment in the AcbC. This difference might be related to the technique, since in the study by Mitchell and Gratton they used chronoamperometry, detecting fast, phasic DA release, whereas in our study we employed microdialysis, which allows measuring more sustained changes in the neurotransmitter content.

Male chemosignals were able to induce an increase in DA efflux in both divisions of the Acb of female rats, although we found a different time course of the dopaminergic response. Thus, DA efflux in the AcbC was significantly higher than baseline already 20 min after the exposure to male chemosignals and remained higher than baseline for the whole experiment, 100 min after the male chemosignals were removed. By contrast, the increase in DA levels in the AcbSh returned to baseline 90 min after the removal of the male chemosignals. Although the maximum increase in DA efflux was similar in the AcbC and AcbSh, i.e., around 30% with respect to baseline (see **Supplementary Figure 2**), the more sustained effect in the AcbC might be related to conditioning processes, whereas the increase in AcbSh could be related to novelty and consummatory responses, as suggested by previous evidence (Bassareo et al., 2002; Cacciapaglia et al., 2012). It should be noted that presenting a new dish with clean bedding also produced an increase in DA release in the AcbC, but this increase was significantly lower than the increase induced by male chemosignals. In addition, it is well established that not only reinforcing, but also aversive and novel stimuli, induce DA efflux in the Acb (Rebec et al., 1997; Horvitz, 2000; McCutcheon et al., 2012).

In this complex panorama, dopaminergic activity in the mesolimbic pathway has been linked to learning through prediction of the rewarding outcome (Schultz, 2002), to the signaling of the incentive motivational properties of reinforcing stimuli (Berridge and Robinson, 1998) and to behavioral activation (Salamone and Correa, 2012). Although it is out of the scope of this study to contribute to the debate on the role of DA to these processes, it could be speculated that the release of DA in the Acb elicited by male chemosignals could be involved in the motivational process enabling pheromone-seeking behavior. If this were the case, blocking DA transmission might blunt the preference for male chemosignals. Previous studies seem contradictory on that point. On the one hand, systemic DA antagonists did not affect the preference of female mice for male-soiled bedding (Agustín-Pavón et al., 2007), whereas DA agonists induced a decrease in preference for unreachable opposite-sex subjects in female rats and male mice (Landauer and Balster, 1982; Ellingsen and Ågmo, 2004). On the other hand, selective 6-OHDA lesions of the anteromedial Acb plus OT disrupted the preference of female mice for male chemosignals (DiBenedictis et al., 2014), although similar lesions failed to affect this preference (Martínez-Hernández et al., 2012). This latter discrepancy might be related to differences in the protocol used, since in the study by Martínez-Hernández et al. (2012) the control stimulus was clean bedding, a stimulus with low incentive value, whereas in the study by DiBenedictis et al. (2014), they used female chemosignals as control. If, on the other hand, DA is related to the effort that an animal has to put to obtain the reinforcing stimulus, the effect of manipulation of the dopaminergic system would only be discovered in tests requiring an effort to obtain the reinforcing chemosignals, which is not the case in our tests, where male chemosignals are readily available. To investigate these possibilities, it would be interesting to carry out future experiments exploring whether the release of DA is correlated with preference or instrumental responses directed to obtain the reinforcing chemosignals.

### Dopaminergic Levels in the Acb Are Modulated by Opioid and Glutamate Antagonism

A final question that we sought to address in our study was related to the pharmacological modulation of the dopaminergic response to male chemosignals. Our results show that locally blocking opioid receptors in the AcbC by naltrexone resulted in a higher increase of DA in AcbC 40 min after termination of the exposure to male chemosignals until the end of the experiment, i.e., the levels of DA were not affected by naltrexone during and immediately after exposure to male-soiled bedding. This delayed effect of naltrexone might be related to the pharmacological profile of this drug, which is both a μ- and κ-opioid receptor antagonist. In this sense, it has been shown that κ-opioid inhibition increases DA efflux in the Acb (Spanagel et al., 1992); therefore, the delayed increase in DA levels might be caused by the pharmacological action of naltrexone over κ-opioid receptors, which would act further disinhibiting the significant rise in DA levels provoked by male chemosignals. By contrast, DA efflux during exposure to clean bedding was unaffected by naltrexone. It is likely that we did not observe this disinhibition due to a more moderate increase in DA efflux elicited by clean bedding.

This result fits into a scenario in which inhibitory opioidergic receptors might be located presynaptically in the excitatory afferents promoting DA efflux and/or in the dopaminergic terminals, so the administration of an opioidergic antagonist would prevent an opioid-mediated termination of the DA release induced by male chemosignals. Since naltrexone failed to affect the DA efflux during the exposure to male-soiled bedding, it is likely that the opioidergic modulation takes place way after the initial increase in DA efflux elicited by the sensory stimulus, to help returning the DA levels to baseline.

To the best of our knowledge, there are only scarce detailed descriptions of the distribution of opioid receptors in AcbC to support this hypothesis. Regarding this, μ-opioid receptors have been described in axon terminals in the AcbSh, providing a possible site of action for naltrexone in disinhibiting the DA efflux. However, previous results showed that activation of opioidergic receptors in AcbC enhances, rather than decreases, extracellular levels of DA, presumably by activation of presynaptic receptors on local inhibitory neurons in the Acb, or by inhibition of GABA projection neurons to the VTA (Hipólito et al., 2008). It should be noted that in that latter study, DA levels were measured in response to an application of agonists of μ- and δ-opioid receptors, whereas in the present study we measured DA efflux after olfactory stimulation. Further studies characterizing in detail the location of opioid receptors in the AcbC are needed to provide anatomical grounds to our result. Finally, our result seems contradictory with the study by Mitchell and Gratton (1991) in male rats showing that systemic treatment with the opioidergic antagonist naloxone attenuated the dopaminergic release in the AcbC induced by female odors. This discrepancy might be related to the site of action of opiates, which is ubiquitous and cannot be determined by using a systemic approach.

As we pointed out above, the increase of DA efflux in the Acb of female rats elicited by male chemosignals might be due to the activity of glutamatergic projections from the amygdala conveying olfactory and vomeronasal information to the Acb rather than to the activity of VTA cells. In fact, afferent activity from the BLA increases DA efflux in the Acb (Floresco et al., 2001a), even in the absence of activation of the VTA neurons, e.g., after inactivation of VTA with lidocaine (Howland et al., 2002). If this were the mechanism by which DA is released by exposure to sexual chemosignals, then pre-synaptic opioid receptors in the glutamatergic afferents could blunt the dopaminergic response and, conversely, antagonists could enhance the DA efflux. In addition, the regulation of DA efflux in the Acb by glutamate is dependent on NMDA receptors (Floresco et al., 2001a; Howland et al., 2002), which are located in presynaptic TH-positive axons (Gracy and Pickel, 1996). Thus, we expected that the administration of kynurenic acid, an antagonist of this type of receptors, would blunt the DA increase. In agreement with our hypothesis, kynurenic acid completely blocked the release of DA elicited by male chemosignals, and even lowered the DA levels with respect to baseline. Further experiments are necessary to test the validity of our hypothesis and to investigate whether activation of the glutamatergic projections from the vomeronasal amygdala are able of to elicit the same DA response in the absence of VTA stimulation.

Finally, kynurenic acid administration in AcbSh via retrodyalisis decreased the overall time spent by female rats exploring male-soiled bedding as compared to vehicle-treated females. Strikingly, the investigation of male bedding was unaffected by the treatment for the first 5 min of the test. Although the two-choice tests and the microdialysis experiments are not comparable, it should be noted that those first 5 min represent the same time window for both type of experiments, when the rats are faced for the first time with male chemosignals. Thus, we wonder whether the initial preference response to male chemosignals is independent on dopaminergic signaling, a possibility which would fit the lack of effect of dopaminergic antagonists on preference for sexual chemosignals reported by Agustín-Pavón et al. (2007) and of VTA and Acb lesions reported by Martínez-Hernández et al. (2006, 2012), but would contradict the results by DiBenedictis et al. (2014). In this scenario, DA efflux to the Acb might be more related to persistent pheromone-seeking behavior than to innate preference for male chemosignals. In fact, kynurenic acid decreased investigation of male chemosignals after the initial 5 min of exposure.

However, an alternative explanation might be that kynurenic acid would reduce the investigation of chemosignals acting over glutamatergic receptors in striatal neurons. Given that DA efflux seems to be caused by chemosignal exposure, then blocking the exposure would prevent the increase in DA levels. To test these possibilities, further experiments should check the effect of blocking DA efflux to the Acb in preference tests and/or instrumental paradigms requiring an effort to gain access to sexual chemosignals, as suggested above. For example, it might be interesting to check whether optogenetic signaling of the amygdalar inputs into the Acb would disrupt the DA efflux and preference for male chemosignals.

Accumulated evidence suggest that the Acb participates in gating the impact of the sensory input on behavior, acting as a limbic-motor interface, and participates in controlling the motivation of an animal to get a reward or the effort that an animal is ready to allocate to obtaining or avoiding a particular stimulus (Salamone and Correa, 2012). Our finding that blocking DA efflux in the AcbSh reduces the investigation of male chemosignals in long exposures (40 min), but not during the first minutes of exposure is consistent with that interpretation.

In summary, our results show that olfactory/vomeronasal information, and in particular sexual chemosignals, are able to elicit a dopaminergic response in the Acb of female rats, and that blocking the release of DA in the AcbSh with a glutamate antagonist reduces the investigation of male chemosignals after the initial exposure. Further experiments should aim to investigate whether the dopaminergic response can be elicited by direct activation of the projections of the vomeronasal amygdala to the Acb, and further explore the role of other divisions of the ventral striatum, in particular the OT, which might be key to control behavior elicited by emotionally salient odors (Agustín-Pavón et al., 2014; DiBenedictis et al., 2014, 2015; Fitzgerald et al., 2014). The use of these olfactory/vomeronasal stimuli can provide an ethologically relevant approach to explore how the brain codes motivation and reward-directed behavior.

## Author Contributions

AP, EL, FM-G, and LG designed research. MJS-C, AO, and LH performed research. MJS-C, AO, TZ, and CA-P analyzed data. MJS-C and CA-P wrote the paper. All authors revised the final version and approved the manuscript.

## Conflict of Interest Statement

The authors declare that the research was conducted in the absence of any commercial or financial relationships that could be construed as a potential conflict of interest.
