# Supplementary material for: Glutamate and Opioid Antagonists Modulate Dopamine Levels Evoked by Innately Attractive Male Chemosignals in the Nucleus Accumbens of Female Rats
Source: Front Neuroanat. 2017 Feb 23;11:8. doi: 10.3389/fnana.2017.00008 (PMC5322247; doi:10.3389/fnana.2017.00008)
Supplement: Supplementary file 1 [file Presentation_1.pdf]

Glutamate and Opioid Antagonists Modulate Dopamine Levels Evoked by Innately Attractive Male Chemosignals in the Nucleus Accumbens of Female Rats

María-José Sánchez-Catalán, Alejandro Orrico, Lucía Hipólito, Teodoro Zornoza, Ana Polache, Enrique Lanuza, Fernando Martínez-García, Luis Granero and Carmen Agustín-Pavón

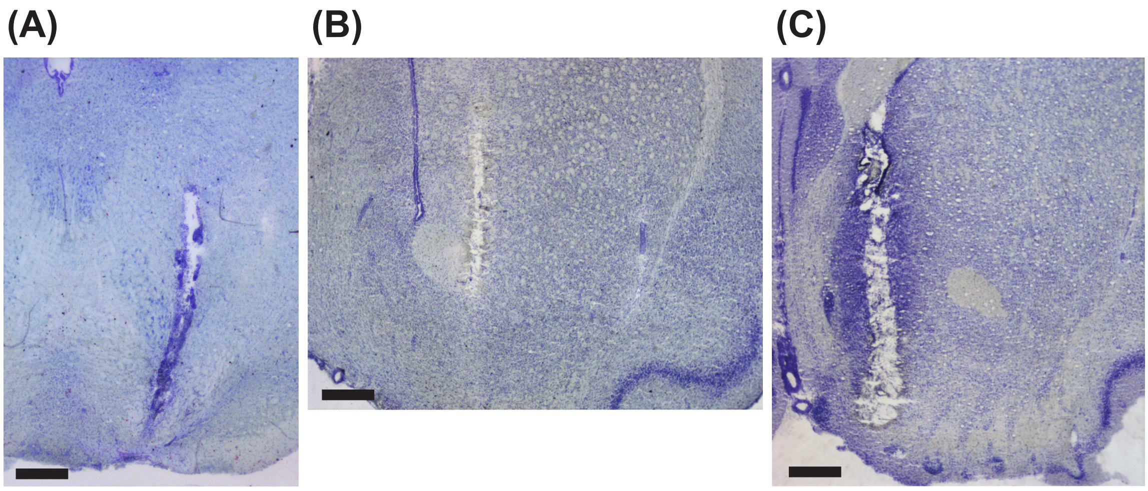

Figure 1: Microphotographs of coronal brain sections through the VTA (A) and Acb (B, C) showing the cannula placement in the pVTA (A) and microdyalisis probe placements in AcbC (B) and AcbShb(C). Scale bar, 500 µm.

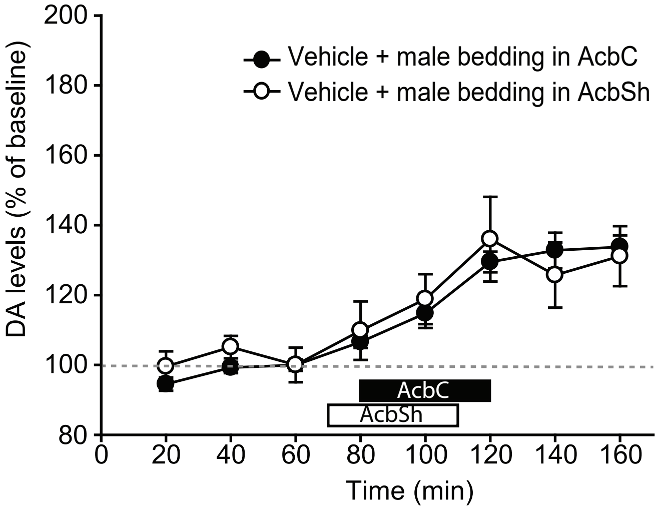

Figure 2: Exposure to male chemosignals induces a maximum increase in DA efflux similar in the AcbC and AcbSh. The DA increase was around 30% with respect to baseline in both Acb subregions. The black (AcbC) and white (AcbSh) bars indicate the period of the bedding exposure. Data are represented as mean ± SEM.
